# Transcriptome Related to Avoiding Immune Destruction in Nasopharyngeal Cancer in Indonesian Patients Using Next-Generation Sequencing

**DOI:** 10.31557/APJCP.2020.21.9.2593

**Published:** 2020-09

**Authors:** Risky Hiskia Poluan, Digdo Sudigyo, Gisti Rahmawati, Dicka Wahyu Setiasari, Salsabila Luthfi Sesotyosari, Tirta Wardana, Indwiani Astuti, Didik Setyo Herianto, Sagung Rai Indrasari, Cita Herawati, Afiahayati, Sofia Mubarika Haryana

**Affiliations:** 1 *Study Program of Biotechnology, Universitas Gadjah Mada, Yogyakarta, Indonesia. *; 2 *Faculty of Medicine, Public Health and Nursing, Universitas Gadjah Mada, Yogyakarta, Indonesia. *; 3 *Universitas Jenderal Soedirman, Central Java, Indonesia. *; 4 *Dharmais Cancer Hospital, Jakarta, Indonesia. *; 5 *Department of Computer Science and Electronics, Universitas Gadjah Mada, Yogyakarta, Indonesia. *

**Keywords:** Nasopharyngeal cancer, transcriptomic, next-generation sequencing, avoiding immune destruction

## Abstract

**Objective:**

This study aims to obtain the transcriptomes profile associated with avoiding immune destruction from nasopharyngeal cancer patients in Indonesia using next-generation sequencing.

**Methods:**

The samples are divided into two types of samples; 1) biopsy of nasopharyngeal cancer tissue samples, 2) brushing tissue of people without nasopharyngeal cancer as control samples. The sequencing results were mapped (HISAT2) and quantified (HTSeq) for differential expression analysis using edgeR software. Transcripts data analyzed with Pantherdb and DAVID software to find genes related to the immune system and pathways related to immune destruction by cancer.

**Results:**

The differential expression results show that 2,046 genes that have a significant differential expression. The 90 genes expression has down-regulated and 1,956 genes expression up-regulated, there are 20 genes related to the immune system. The 20 genes related to the immune system by analyzing lionproject.net that directly related to hallmark avoiding immune destruction that genes are *CXCL9/10/11*. The gene expression of *CXCL9/10/11* regulates *PD-L1* expressions via the Jak/STAT signaling pathway. The interaction between the extracellular domain PD-1 and PD-L1 in cancer cells have avoiding immune destruction.

**Conclusion:**

The results of this study suggest that the gene expression of CXCL9/10/11 have up-regulated is related to avoiding immune destruction that can use as an early detection biomarker of nasopharyngeal cancer in Indonesian patients.

## Introduction

According to data from the World Health Organization (WHO) in 2015, cancer is the first or second cause of human death in the age before 70 years in 91 countries from 172 countries (Bray et al., 2018). Worldwide, nasopharyngeal cancer is cancer with a rare incidence of malignancy, with an incidence rate generally <1 per 100,000 people per year, but areas with high incidence are found in the southern regions of China, Southeast Asia, North Africa, and the Arctic (Ferlay et al., 2013). In southeast Asia for example in Indonesia, nasopharyngeal cancer is a major problem because there are 15,000 new cases per year (Ferlay et al., 2008).

Based on data obtained from the Global Cancer Observatory (GCO), the number of cases of nasopharyngeal cancer in men in Indonesia on ranked fourth. Estimated that the incidence of nasopharyngeal cancer is 12,000 new cases per year, with all cases related to Epstein Barr virus (EBV) (Adham et al., 2012).

In Indonesia, 100% of five-year-old children are infected with EBV and carry latent viruses for life. Early-stage nasopharyngeal cancer (NPC) is difficult to diagnose clinically because of its hidden location in the nasopharynx (Adham et al., 2012). Nasopharyngeal cancer shows high metastatic rates, tend to be present at an advanced stage (clinical stages III and IV) when diagnosed. More than 70% of patients are at an advanced stage when diagnosed at the clinic (Lo et al., 2004; Tang et al., 2016).

Nasopharyngeal cancer has a complex etiology. Nasopharyngeal cancer has a poor prognosis due to late presentation of the lesion, lack of knowledge in molecular mechanisms, no effective markers for early detection, and poor response to currently available therapies, it has been reported (Janvilisri, 2015). Patients with nasopharyngeal cancer can recur within two years. Almost half of the total patients who have treatment are considered very risky to relapse (Li et al., 2017). One key aspect of cancer research is to understand the principles and mechanisms of changes in gene expression that contribute to carcinogenesis and cancer development. In the post-genomic era, many methods for profiling gene expression have been developed, such as microarrays and next-generation sequencing, genomically, miRNA-omics, transcriptomics, proteomics and, metabolomics, to classify cancers and identify new biomarkers for prognosis and diagnosis, and as targets for therapy (Bucca et al., 2004).

One strategy to identify the main patterns of expression, next-generation sequencing (NGS) has been used to explore genetic heterogeneity and gene expression through transcriptomes profiles related to cancer development. In tongue and mouth cancer, avoiding immune destruction is the main transcriptomic characteristic associated with the invasive and metastatic development of tongue and mouth cancer. Next-generation sequencing (NGS) as a method of high throughput gene expression analysis as a resource to investigate large changes in cancer cell expression patterns (Pérez-Valencia et al., 2018).

There is no effective target therapy for advanced nasopharyngeal cancer so far. The scarcity of transcriptome data for nasopharyngeal cancer obstructs the biological understanding of nasopharyngeal cancer development. Using transcriptomic data analysis is expected to successfully identify the evaluation of gene expression levels/transcriptomes. Transcriptomic data can be used as new prognostic and diagnostic markers for cancer, particularly related to hallmark avoiding immune destruction in Indonesian nasopharyngeal cancer patients.

## Materials and Methods

*Sample Collection and Ethical Clearance*

Ten samples from the biopsy of nasopharyngeal cancer tissue and six samples from the brushing tissue of people without nasopharyngeal cancer. Samples obtained from clinic ENT-HN of Sardjito Hospital Yogyakarta and Dharmais Cancer Hospital Jakarta. The sampling technique in this study is a consecutive sampling. The technique to get samples is by consecutive sampling, which is looking for patients who meet the inclusion and exclusion criteria until the required number of samples is met. Ethical clearance was obtained from the Ethics Commission of the Faculty of Medicine, Public Health, and Nursing at Universitas Gadjah Mada with Reference Number: KE/FK/0250/EC/2019.

*Isolation of Total RNA*

Isolation of total RNA from the samples used the Qiagen RNeasy Mini Kit Protocol. Total RNA isolation begins from extraction RNA to produce RNA purification. The total RNA isolation process is carried out in an Integrated Research Laboratory, Faculty of Medicine, Public Health, and Nursing of Universitas Gadjah Mada.

*Library Preparation*

Library preparation using the TruSeq Stranded Total RNA Sample Prep LS protocol.

*Cluster Generation and Sequencing*

Cluster generation and sequencing occur in next-generation sequencer the NexSeq 550 system using the Illumina protocol. The sequencing process carried out in an Eijkman Institute for Molecular Biology, Jakarta, Indonesia.

*Bioinformatics Analysis*

**Figure 1:**
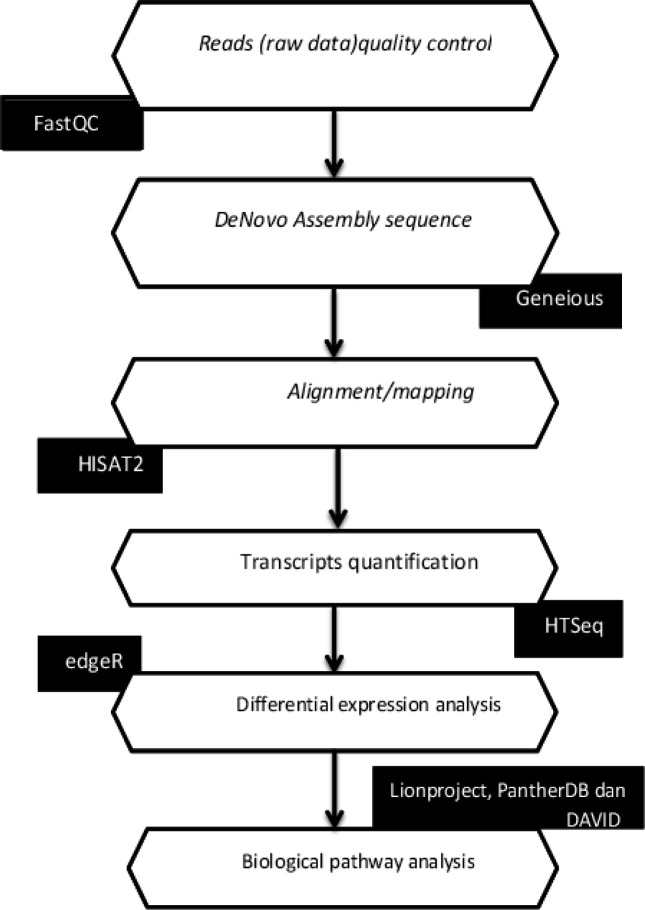
Bioinformatics Pipeline for Transcriptomic Analysis (RNA-Seq)

Bioinformatics analysis used several tools for interpreting data from raw reads to expression differences of each gene analyzed (Figure 1).

*Processing reads*

Results of RNA sequencing in fasta format. Quality control (QC) of fasta as raw reads of transcripts to validate the transcripts. Software to test quality control in the transcripts is fast QC (http://www.bioinformatics.babraham.ac.uk/projects/fastqc/).

*DeNovo Assembly Sequence*

RNA sequencing results consist of reads1 and reads 2. RNA Sequencing in the form of reads 1 and reads 2 will be merged using de Novo assembly on Genious Software. Alignment and mapping reads can be analyzed if they are merged.

*Alignment/Mapping*

The HISAT2 will alignment/mapping of transcripts to the reference index, the reference index is H. Sapiens, UCSC hg38. The transcripts processed using HISAT2 software are operated using Linux OS on the High-Performance Computer in the Department of Computer Science and Electronics Universitas Gadjah Mada

*Quantification of the Number of Transcripts*

Transcripts from HISAT2 results be used to count transcripts using HTSeq and are operated on LINUX OS.

*Analysis of Differences in Gene Expression Levels*

Differences in gene expression levels between control samples and nasopharyngeal cancer analyzed by using R/Bioconductor package edgeR (version 2.6.10).

*Biological Reaction Pathway Analysis*

Panther^®^ (http://pantrherdb.org) to determine the biological processes of specific genes based on gene ontology and biological pathways. Hallmark of cancer-related to avoiding immune destruction analyzed by lionproject.net.

## Results

*Validation of Total RNA Isolation*

Total RNA Isolation validated by Nanodrop and Qubit tool. Nanodrop is used to measure the purity of RNA and the Qubit is used to measure the concentration of RNA. Measuring the purity of RNA with Nanodrop is between 1.7 - 2.1 nm with A260/A280 wavelength. The measuring of RNA concentration with Qubit is about 10ng / µL (Table 1). Based analysis of Nanodrop and Qubit has obtained 16 samples that fit the criteria for library preparation.

*cDNA Library*

**Figure 2:**
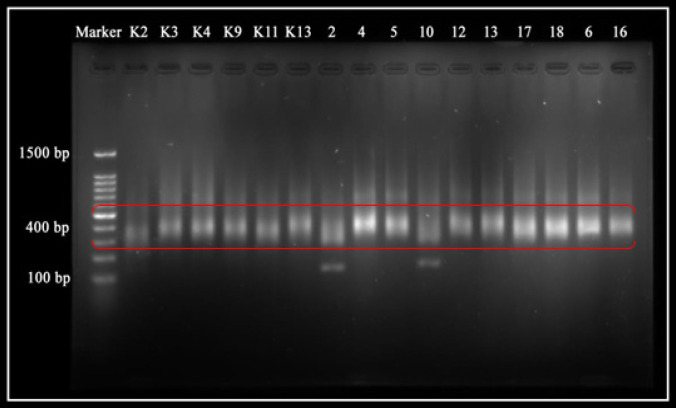
Gel Electrogram of cDNA Fragments. Control samples (K2, K3, K4, K9, K11, K13) and nasopharyngeal cancer samples (2, 4, 5, 6, 10, 12, 13, 16, 17, and 18). The cDNA fragment size is 400 bp (☐).

The results of library preparation are the cDNA library. The results of electrophoresis as quality control of cDNA (Figure 2) has 16 samples that have the same fragment size. Based on Electrophoresis Gel results from control samples, namely K2, K4, K9, K11, K13, and nasopharyngeal cancer samples, namely 2, 4, 5, 6, 10, 12, 13, 16 and 17. All samples could detect in a band with a fragment size of 400 bp. 

The cDNA concentration has normalized. Based on the test quantity of concentration using qPCR from 16 samples. The nine samples were obtained from the normalized concentration using qPCR. The nine samples are K2, K4, 4, 5, 6, 10, 12, 13, and 16. Normalization is a process to uniform the concentration of each sample that determines the eligibility of a sample to be sequenced with Next-generation sequencer.

*Quality Control of NGS Results*

Data from NGS results tested for good quality using fast QC. The sample of K2 and K4 as control samples (Non-NPC) and samples 4, 5, 6, 10, 12, 13, and 16 as samples of nasopharyngeal cancer (NPC) have good quality (Table 2),, this is indicated by a score of zero (0) in the poor quality column, long sequence, and GC Content. The total sequence of each sample is between 18128296 bp (in sample 10) to 169898666 bp (in sample K4). The sequence length of each sample is between 35 and 76. The percentage of GC (guanine-cytosine content) is between 48 and 58. The indicator of poor quality with a score of 0 and the percentage of GC as an indicator of the average human genome that is between 35 to 60 indicates that the sample is good quality.

RNA-seq from NGS results is obtained in the form of a fasta file. Fasta file which has been in De Novo assembly with Geneious makes reads 1 and reads 2 merged (Table 3). 

*Analysis of differential expression*

RNA-seq from NGS results called transcripts. Analysis of differential expression begins with transcripts or reads is alignment to the reference genome to identify the position of the genome analyzed based on the reference index is H. Sapiens, UCSC hg38 using HISAT2.

The results of count transcripts using HTSeq and annotations obtained 60617 of transcripts. Data from the annotation were filtered and normalized to transcripts that had many copies less than 10. Data from the annotation were filtered and normalized as many as 25943 transcripts.

**Figure 3:**
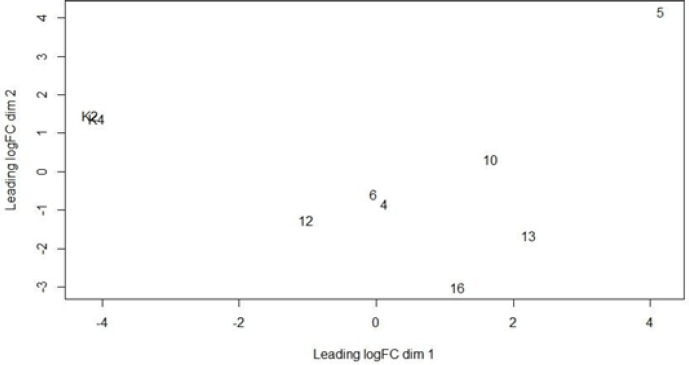
Plot Multidimensional Scaling (MDS) Using edgeR. Dimension Matrix between Control Samples and Nasopharyngeal Cancer Samples. The K2 and K4 samples are the same dimensions because they coincide while the nasopharyngeal cancer samples 4, 5, 6, 10, 12, 13, and 16 appear to be spreading out and away from the dimensions of the control sample. This confirms that control samples (K2 and K4) differed in groups from nasopharyngeal cancer samples (4, 5, 6, 10, 12, 13, and 16)

The Plot Multidimensional scaling (MDS) using edgeR (Figure 3) of the control sample from K2 and K4 samples are in the same plot. The control samples indicate that the sample data in the same matrix. But for nasopharyngeal cancer sample data 4, 5, 6, 10, 12, 13, and 16 is dispersed. The matrix indicates that the control sample and nasopharyngeal cancer sample are in different characters.

**Figure 4:**
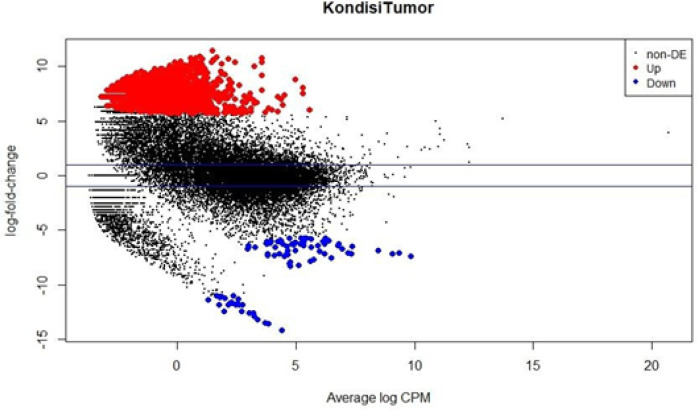
Plot Biological Coefficient of Variation (BOV) Using edgeR. Samples with red dots (•) are expressions of genes that are significantly up-regulated. The blue dots (•) are expressions of genes that are significantly down-regulated. The black dots (•) are expressions of genes that significantly do not significant changes in nasopharyngeal cancer samples compared with control samples

On the Plot Biological Coefficient of Variation (BVC) using edgeR, the red plots are the genes expression with significantly up-regulated, while the black plots are the genes expression that has not changed significantly, and the blue plots are significantly down-regulated genes expression (Figure 4). Genes that significantly up-regulated expression are more numerous than down-regulated expression genes.

Changes in gene expression based on comparison of sample control and control of nasopharyngeal cancer using edger analysis. Based on p-value <0.005 and logFC (fold change expression of transcripts), 25,943 genes or transcripts were obtained that had changes in expression. Total number of genes expression that was down-regulated as many as 90 genes, 23,897 genes expression that did not significant changes, and the total number of genes expression that up-regulated expression as many as 1,956 genes.

Based on p-values less than 0.05 on edger analysis, 90 genes have downregulated expression and 1,956 genes that have up-regulated expression. 2,046 genes have significant changes in gene expression. The gene symbol of this total genes is input to pantherdb.org to see the biological pathways (supplementary 1).

The PANTHER classification system (http://www.pantherdb.org) is a comprehensive system that combines genomes, classification of gene functions, pathways, and statistical analysis tools that allow researchers to analyze large-scale genomic experimental data (Mi et al., 2013).

Twenty genes related to the immune system were obtained from Pantherdb analysis (supplementary 1). Through this analysis, protein classes can be obtained from genes related to the immune system based on the analysis of biological processes (supplementary 2).

*The Down-regulated Genes*

The down-regulated genes expression is ALOX15 and HMGB4 (Table 4). The expression gene of *ALOX15 *(15-lipoxygenase-1) plays an important role in the formation of lipid mediators (e.g Lipoxin and Resolvin) to stop inflammation. *ALOX15*’s gene expression is down-regulated in colorectal cancer (CRC). Gene expression of *ALOX15* suppresses the TNF-α signaling pathway, IL-1β/NF-κB, and IL-6/STAT3, which play a major role in increasing colorectal cancer through chronic inflammation (Tian et al., 2017). Defining the role of gene expression of *ALOX15* regulation can identify molecular regulation that can be targeted to suppress the promotion of tumorigenesis through chronic inflammation. Compared to this study, the *ALOX15* gene expression has been down-regulated so that it is possible that gene expression *ALOX15* whose role as an inflammatory and cancer suppressor. The hypothesis that the *ALOX15* gene expression is down-regulated in the development of nasopharyngeal cancer can be presumed to be the same regulation. *HMGB4* gene expression was down-regulated through the development of liver cancer (Zhang et al., 2018). Consistent with this study that the *HMGB4 *gene expression has been down-regulated in nasopharyngeal cancer patients in Indonesia.

*The Up-regulated Genes*

The genes that have up-regulated expression changes, namely *HEPACAM, BTNL3, REG3A, REG1B, DPP6, REG1A, CXCL11, CLEC6A, GGT1, CXCL9, CRYAB, HSPB6, FAM43B, PGLYRP3, PGLYRP2, COLEC12, PLA2G2F, dan CXCL10* (Table 4).

*Analysis of Biological Pathways*

**Figure 5:**
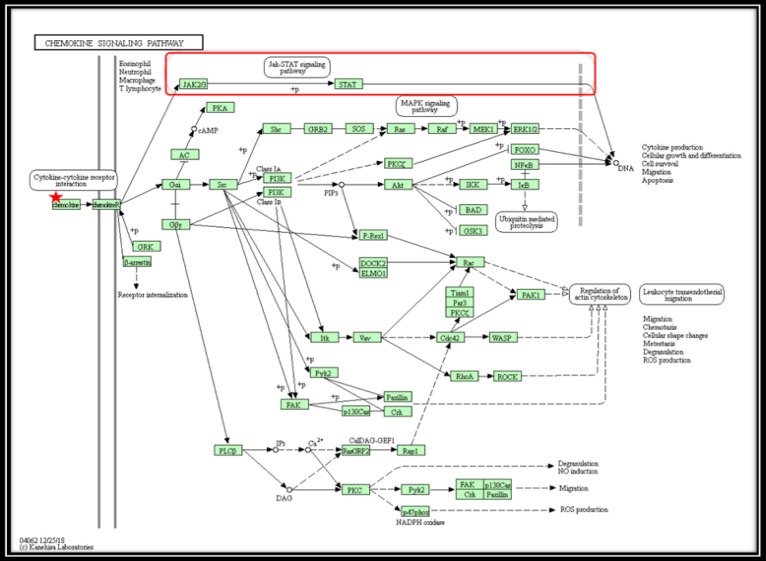
Chemokine Signaling Pathway Based on DAVID Analysis of KEGG Pathway Database. The CXCL9/10/11 protein is the CXC chemokine class. The CXC chemokine class is a subfamily of the Chemokine family. The *CXCL9/10/11* genes are selective ligands for *CXCR3. CXCL9/10/11-CXCR3 *gene expression can regulate PD-L1 expression by activating the Jak/STAT (□) pathway related to immune evasion from T cells

Twenty genes related to the immune system analyzed by the lionproject.net tools to get genes related to avoiding immune destruction. The genes related to immune destruction obtained through the lionproject.net tool namely *CXCL9, CXCL10, CXCL11, CRYAB, PGLYRP2, *and *PGLYRP3* (Supplementary 3). The expression of genes related to avoiding immune destruction (AID) was analyzed by the DAVID Bioinformatics Database. Analysis of the large gene list is an exploratory and computational procedure. Compared to other similar services, DAVID provides several unique features and capabilities, such as an integrated and extensive back-end annotation database, sophisticated module enrichment algorithms and, strong exploration capabilities in an integrated data mining environment (Huang et al., 2009). The pathway was obtained from 3 genes namely the genes *CXCL9, CXCL10, *and *CXCL11* (supplementary 4). Analyzed using the David Bioinformatics Database tool to obtain pathways related to avoiding immune destruction based on gene *CXCL9/10/11* (Figure 5).

*The Expression Genes CXCL9, CXCL10 And, CXCL11 Related to Avoiding Immune Destruction*

**Figure 6:**
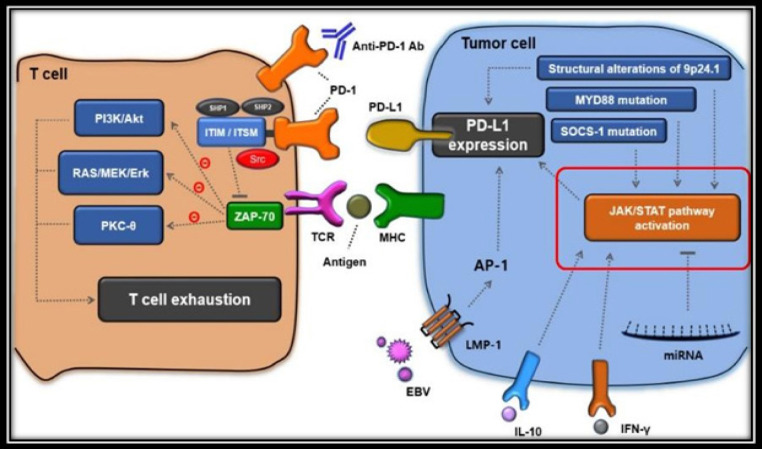
Immune Evasion Mechanisms Associated with the PD-1/PD-L1 Signaling Pathway (Song et al., 2019). PD-1 is expressed on the surface of T cells that are activated by downstream T cell receptor signaling (TCR), with the important function of checking points in regulating the T-cell mediated immune response. PD-1 provides a signal inhibition to regulate T-cell activation after binding to the PD-L1 ligand because the Jak/STAT (□) pathway is up-regulated. The interaction between the extracellular domain PD-1 and PD-L1 induces changes in the conformation of PD-1 that produce phosphorylation of the cytoplasmic ITIM and ITSM, which then recruits Src Homology Region 2-Containing Protein Tyrosine Phosphatase-2 (SHP-2) and SHP-1. After SHP-1/2 is recruited, the interaction dephosphorylates protein - ζ associated protein 70 (ZAP70) protein as a downstream member of the TCR signaling pathway and thereby inhibits the phosphatidylinositol-3- kinase/Akt (PI3K/Akt) pathway, RAS/MEK/Erk, and protein kinase Cθ - (PKC-θ). Finally, the inhibitory pathway mediated by PD-1 is closely related to decreased T-cell proliferation and decreased IL-2 production, and promotes T-cell apoptosis, which causes T-cell fatigue (Song et al., 2019).

*CXCL9/10/11 *is a gene that produces CXC chemokine class proteins. The CXC chemokine class is a subfamily of the chemokine family. Chemokines (asterisks) are a class of functional chemotactic peptides that contribute to some tumor-related processes (Figure 6). During the occurrence and development of cancer cells, this chemokine class is often accompanied by a series of molecular and biological changes (Zhu et al., 2012). The *CXCL9/10/11* gene is a selective ligand for CXCR3. Ligands are usually expressed at low levels in homeostatic conditions but are regulated by cytokine stimulation. 

Chemokine will bind to the ligand.* CXCL9/10/11* gene expression with up-regulated CXCR3 affected the Jak/STAT pathway. the study of earlier through bioinformatics analysis found that the expression of the *CXCL9/10/11-CXCR3* gene can regulate *PD-L1* expression by activating the Jak/STAT pathway (Zhang et al., 2018).

Based on the signaling pathway and immune evasion mechanism, the expression of the up-regulated *CXCL9/10/11-CXCR3* gene makes the Jak/STAT signaling pathway regulates PD-L1 in cancer cells. The interaction between the extracellular domain PD-1 and PD-L1 is thought to make cancer cells avoiding immune destruction of immune cells especially T cells.

**Table 1:** RNA Purity and Concentration

No.	Control (K) and NPC (Number) Samples	RNA Purity A260/A280 (nm)	RNA Concentratiom Ng/µL
1	K2	1,905	42,8
2	K4	1,877	73,0
3	K9	1,917	61,0
4	K11	1,752	59,0
5	K13	1,908	20,9
6	K3	1,965	19,7
7	4	1,931	9,86
8	5	1,871	15,1
9	6	1,89	31,3
10	10	1,997	100
11	12	1,846	15,4
12	17	1,970	5,63
13	2	1,859	8,91
14	16	2,04	32,4
15	13	2,21	26,9
16	18	2,217	26

**Table 2:** Normalization Concentration of qPCR

No	Samples	cDNA Concentration	cDNA fragment (bp)	Information
1	K2	9.18	400	Non-NPC
2	K4	5.23	400	Non-NPC
3	4	4.73	400	NPC
4	5	11.82	400	NPC
5	10	12.77	400	NPC
6	12	6.28	400	NPC
7	13	9.65	400	NPC
8	6	14.44	400	NPC
9	16	27.61	400	NPC

**Table 3:** Fast QC of Sequencing Results

No	Samples Code	Reads	Total sequence (bp)	The sequence with poor quality	Sequence length	%GC
1	K2	1	33097617	0	35-76	53
		2	33097617	0	35-76	55
2	K4	1	169898666	0	35-76	50
		2	169898666	0	35-76	51
3	4	1	186979159	0	35-76	57
		2	186979159	0	35-76	58
4	5	1	20413210	0	35-76	56
		2	20413210	0	35-76	57
5	6	1	36803557	0	35-76	53
		2	36803557	0	35-76	53
6	10	1	18128296	0	35-76	49
		2	18128296	0	35-76	52
7	12	1	87652547	0	35-76	56
		2	87652547	0	35-76	58
8	13	1	67777774	0	35-76	53
		2	67777774	0	35-76	54
9	16	1	64223909	0	35-76	48
		2	64223909	0	35-76	49

**Table 4:** Up-Regulated and Down-Regulated Genes Related to the Immune System

No.	Gene symbol	Gene level expression	logFC	pValue
1.	*HEPACAM*	Up regulated	7.03	0,000861076
2.	*BTNL3*	Up regulated	6.175	0,003126123
3.	*REG3A*	Up regulated	8.235	0,000221588
4.	*REG1B*	Up regulated	7.24	0,001220327
5.	*DPP6*	Up regulated	8.564	0,000128259
6.	*REG1A*	Up regulated	7.822	0,00042651
7.	*CXCL11*	Up regulated	5.866	0,003107221
8.	*CLEC6A*	Up regulated	5.885	0,003193691
9.	*GGT1*	Up regulated	6.865	0,001413962
10.	*CXCL9*	Up regulated	6.037	0,002302748
11.	*CRYAB*	Up regulated	6.245	0,002202237
12.	*HSPB6*	Up regulated	8.03	0,000239884
13.	*FAM43B*	Up regulated	8.235	0,000221588
14.	*PGLYRP3*	Up regulated	7.057	0,000794174
15.	*PGLYRP2*	Up regulated	6.294	0,002315018
16.	*CXCL10*	Up regulated	5.834	0,002964692
17.	*COLEC12*	Up regulated	5.785	0,003339016
18.	*PLA2G2F*	Up regulated	6.708	0,001317819
19.	*HMGB4*	Down regulated	-0.808	0,0000954
20.	*ALOX15*	Down regulated	-7.164	0,000628698

Genes have significant changes expression based on p-value <0.005 and logFC: - downregulated, + upregulated

## Discussion

Transcriptomes patterns related to avoiding immune destruction of genes that have significantly changed expression levels, there are eighteen up-regulated genes and, two down-regulated genes are RNA coding. Genes related to immune systems that have changed levels of up-regulated expression are H*EPACAM, BTNL3, REG3A, REG1B, DPP6, REG1A, CXCL11, CLEC6A, GGT1, CXCL9, CRYAB, HSPB6, FAM43B, PGLYRP3, PGLYRP2, COLEC12, PLA2G2F, and CXCL10, CRYAB, HSPB6, FAM43B, PGLYRP3, PGLYRP2, COLEC12, PLA2G2F,* and *CXCL10*, down-regulated *ALG15 *and *CXCL10 *genes. The genes associated with avoiding immune destruction, gene expression *CXCL9/10/11* can regulate PD-L1 expression through STAT. This finding is suggested to be a new mechanism that regulates the avoiding factor of immune destruction through PD-L1. Related to this research, explores transcriptomes patterns only in RNA coding, suggestions for further research so that exploration of transcriptomes patterns can explore non-coding RNA sections to see regulation with mRNA.
